# Mechanical Integrity of Conductive Carbon-Black-Filled Aqueous Polymer Binder in Composite Electrode for Lithium-Ion Battery

**DOI:** 10.3390/polym12071460

**Published:** 2020-06-30

**Authors:** Kehua Peng, Yaolong He, Hongjiu Hu, Shufeng Li, Bao Tao

**Affiliations:** 1 Shanghai Institute of Applied Mathematics and Mechanics, School of Mechanics and Engineering Science, Shanghai University, Shanghai 200072, China; 13437288789@139.com (K.P.); yaolonghe@shu.edu.cn (Y.H.); lishufeng@shu.edu.cn (S.L.); taoboliefe@163.com (B.T.); 2 Shanghai Key Laboratory of Mechanics in Energy Engineering, Shanghai 200072, China

**Keywords:** structure integrity, aqueous polymer binder, conductive carbon black, composite electrode, lithium-ion battery

## Abstract

The mechanical stability of aqueous binder and conductive composites (BCC) is the basis of the long-term service of composite electrodes in advanced secondary batteries. To evaluate the stress evolution of BCC in composite electrodes during electrochemical operation, we established an electrochemical–mechanical model for multilayer spherical particles that consists of an active material and a solid-electrolyte-interface (SEI)-enclosed BCC. The lithium-diffusion-induced stress distribution was studied in detail by coupling the influence of SEI and the viscoelasticity of inorganic-filler-doped polymeric bonding material. It was found that tensile hoop stress plays a critical role in determining whether a composite electrode is damaged or not—and circumferential cracks may primarily initiate in BCC, rather than in other electrode components. Further, the peak tensile stress of BCC is at the interface with SEI and does not occur at full lithiation due to the relaxation nature of polymer composite. Moreover, mechanical damage would be greatly misled if neglecting the existence of SEI. Finally, the structure integrity of the binder and conductive system can be effectively improved by (1) increasing the carbon black content as much as possible in the context of meeting cell capacity requirements—it is greater than 27% and 50% for sodium alginate and the mixtures of carboxy styrene butadiene latex and sodium carboxymethyl cellulose, respectively, for composite graphite anode; (2) reducing the elastic modulus of SEI to less than that of BCC; (3) decreasing the lithiation rate.

## 1. Introduction

With the rapid development of portable electronic devices and electric vehicles, as one of the main choices for energy storage and energy supplied systems, lithium-ion batteries are deemed to be in urgent need of achieving long lifetimes [1,2]. In order to fulfill this, industrial and the scientific sectors have oriented efforts towards understanding their mechanical behavior and the underlying damage mechanisms of battery components from active particles [3,4] to cell structures [5,6]. One has recognized that the primary cause for capacity attenuation of rechargeable lithium batteries is that the Vegard stress induced by the electrochemical reaction cannot only damage the active substances [7], but also polymer bonding materials in the composite electrode [8,9,10]. Therefore, it is extremely significant to study the stress distribution and mechanical stability of the electrode particle–binder system.

Conventionally, polymer materials play two pivotal roles in lithium-ion batteries. They are used as separators to separate positive and negative electrodes, and as adhesives for binding isolated active particles to the current collector [11]. For the former, microporous membranes based on semi-crystalline polyolefin materials such as polyethylene (PE), polypropylene (PP) and their blends are widely used in liquid electrolyte lithium-ion batteries [12,13]. As for all-solid-state batteries, with great efforts made by the scientific community, various poly(ethylene oxide) (PEO)-based solid polymer electrolytes obtained by crosslinking [14,15,16], blending [17,18] or grafting [19] have been applied. For the latter, poly(vinylidene fluoride) (PVdF) are the most commonly used and investigated binding materials mainly because of good electrochemical stability. In the literatures, focusing on the mechanical stability of particle–binder system, Rahani and Shenoy first studied the mechanical degradation in the graphite anode bonded by PVdF using finite element methods. They found that the yield stress level of PVdF determined the average stress of the composite electrode [20]. Takahashi also analyzed the stress evolution of an isolated graphite sphere enclosed by conductive additive filled PVdF in the lithiation process [21]. It was indicated that the polymeric and conductive composite (BCC) had certain constraints on the lithiation deformation of active material, leading to a decrease in the tensile stress of graphite particle. Moreover, the PVdF-based BCC is more likely to take place mechanical degradation in the circumferential direction compared to the active materials. In addition, Singh and Bhandakkar studied the stress evolution of a spherical electrode particle/PVdF system during galvanostatic electrochemical cycling [22]. The viscoelasticity of polymer binders was proven to affect the diffusion induced stress (DIS) in active material and binder and decreasing viscosity and characteristic relaxation time could weaken DIS of the composite electrode. In contrast with above one-dimensional particle models, Higa and Srinivasan calculated the stress of axisymmetric silicon particle sandwiched between two cylinder of PVdF in the course of charging [23]. The simulation disclosed that most of the strain energy of electrode system was stored by PVdF and the energy per interfacial area decreased with particle size and binder stiffness. For this reason, the debonding between active material and BCC may be one of the causes for the degradation of silicon electrode capacity. Lee et al., further investigated the interface failure of a spherical graphite particle and a cylindrical PVdF binder [24]. It demonstrated that the delamination of binders and active particles in the lithiation process was diametrically opposite to the damage mechanism of that inside the active particle. The high lithium-concentration gradient, caused by the large particle size and high charging rate, resulted in the increase of maximum principal stress in the active particles. However, it could help to decrease the interface stress between the binder and electrode material. In consideration of the complex mesostructures of electrode particles and binders, the coupled electrochemical–mechanical simulation was carried out using the experimentally reconstructed microstructure, which was captured by the scanning electron microscope [25] or nanocomputed tomography (X-ray nano-CT) techniques [26]. These works well revealed the roles of electrode geometry of active materials, binder loading and boundary conditions on its surface on the stresses in electrode and PVdF binder under lithiation–delithiation cycling. It is noted that the aforementioned investigations neglected the influence of solid electrolyte interface (SEI) film and conductive agent content in BCC. However, the published reports indicated that SEI formed on the surface of active materials during the lithiation process was strongly associated with the mechanical stability of the electrode material [27] and the conductive additives acted upon a complicated mechanical role when added into the polymer binders for composite electrodes [28,29,30,31,32].

Due to the popular PVdF binder contains toxic organic solvent, sodium alginate (SA) [33,34,35], polyacrylic acid (PAA) [36,37], sodium carboxymethyl cellulose (CMC) [38], styrene–butadiene rubber (SBR) [39], which use water as solution, may be regarded as the potential constitutes in composite electrode for advanced secondary battery. Considerable efforts have been devoted to clarifying the influence of binder nature on the cycle stability and rate performance of cell. Owing to high bonding strength of aqueous polymer binders, high-capacity electrodes also exhibited mechanical stability, good capacity retentions and rate capabilities [36,37]. It is noticed that the cohesion properties of PAA binder acted a pivotal role on the mechanical integrity and electrochemical stability during charging–discharging process. Recently, Li et al., compared the influence of binder stiffness on bending deformation and DIS in Si anodes with SA, Nafion and PVdF with the results suggesting that the binder plays an important role in lithiation-induced deformation and the cracking of composite electrodes [40]. Wang et al., further observed that the elastic modulus and hardness of Si composite electrodes were mainly related to the mechanical properties of water-soluble binders, instead of the adhesion between binders and active particles. These findings may help to understand how the aqueous polymer adhesive system impacts the mechanical stability of electrode materials and vigorously promote the development of high performance and durable composite electrodes. Nevertheless, due to the lack of mechanical properties of conductive agent filled water soluble polymer composite under liquid electrolyte, the fracture mechanism is still not clear for the kind of binder and conductive materials in the composite electrodes. To date, there has been little exploration into the structural integrity of electrode particles system with SEI film, which is enclosed by the binder and conductive composite (BCC).

In this study, we establish an electrochemical-mechanical model for the multilayer spherical particles that consist of an active material, SEI and BCC. The lithium concentration and diffusion induced stress distribution in the electrode system have been emphatically discussed by coupling the effects of SEI and the viscoelasticity of polymer binder. In order to clarify the mechanical failure mechanism of aqueous BCC under realistic condition, the evolution of peak stress in BCC is investigated systematically under different water–based polymer binders, loading of conductive carbon black, elastic modulus and thickness of SEI, as well as charging rates. In contrast to the single-particle or particle-BCC coating structures, we found—possibly for the first time—that the circumferential cracks induced by lithiation may primarily initiate in BCC rather than in other electrode components.

## 2. Model Description

**Figure 1:**
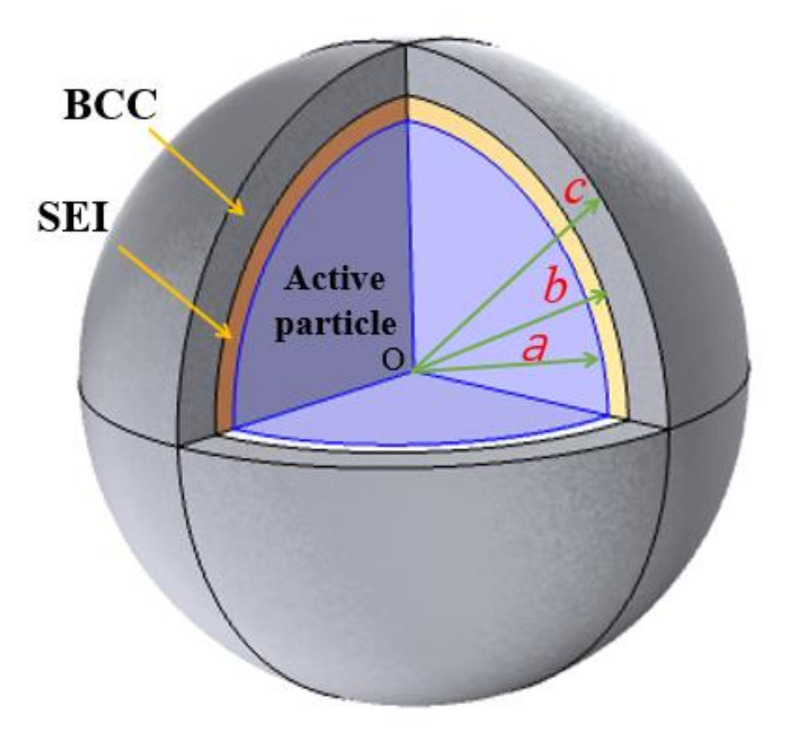
Schematic plot of a multilayer spherical electrode particle system.

The multilayer electrode particle system composed of active material, SEI film and BCC as shown in Figure 1 are considered. It is assumed that the particles are spherical in structure, SEI and BCC are uniformly deposited and coated on them, and the corresponding radii are a, b and c, respectively. Due to high porosity in the graphite electrode composite, the mechanical interaction among the active particles may be negligible. This is to say, there are no external forces on the exposed particle surface. Similar boundary condition at outer surface were adopted for determining the stress evolution of electrode particle system in many published investigations [21,22,23,24]. Therefore, the spherically symmetric particle with free traction at the outer surface of the BCC is applied to depict the composite graphite electrode. Based on the hypothesis, the thickness of SEI is $h_{SEI}=b-a$, while the thickness of BCC is $h_{BCC}=c-b$.

Under the spherical coordinates, the diffusion of lithium in active particles is determined by the following equation:

(1) $$\frac{\partial c}{\partial t}+\frac{1}{r^{2}}\frac{\partial \left(r^{2}J\right)}{\partial r}=0$$

Here, $c\;\left(\mathrm{mol}/m^{3}\right)$ is the molar concentration of lithium and $J=Dc\nabla \mu /\left(R_{g}T\right)$ is the related lithium flux, where$\;D\;\left(m^{2}/s\right)$, $\mu \;\left(J/\mathrm{mol}\right)$, $R_{g}\;\left(J/K/\mathrm{mol}\right)$ and $T\;\left(K\right)$ represent the diffusion coefficient of lithium, chemical potential, universal gas constant and temperature, respectively. Taking the influence of mechanical energy caused by stress on chemical potential into consideration, the chemo-mechanical potential μ can be further expressed as:

(2) $$\mu =\mu_{0}+R_{g}T\ln c-\Omega \sigma_{h}$$

where $\mu_{0}$ is the an invariant reference potential, $\Omega \;(m^{3}/mol)$ is the partial molar volume of lithium and $\sigma_{h}$ is the hydrostatic stress, which can be calculated by radial stress $\sigma_{r}$ and circumferential stress $\sigma_{\theta }$ under the spherical coordinates, i.e., $\sigma_{h}=\left(\sigma_{r}+2\sigma_{\theta }\right)/3$.

The following governing equation of lithium diffusion can be obtained through substituting Equation (2) into Equation (1).

(3) $$\frac{\partial c}{\partial t}=\frac{1}{r^{2}}\frac{\partial }{\partial r}\left\{Dr^{2}\left(\frac{\partial c}{\partial r}-\frac{\Omega c}{R_{g}T}\frac{\partial \sigma_{h}}{\partial r}\right)\right\}$$

Driven by the gradient of chemical potential, the lithiation and delithiation on the surface of the particles are assumed to take place at galvanostatic or potentiostatic conditions and the corresponding initial and boundary conditions are expressed as [41,42,43]:

(4) $$\begin{array}{ll} c=c_{0}\left(t=0\right) & \\ {\left.-n\cdot J\right|}_{r=0}=0,{\left.-n\cdot J\right|}_{r=a}=\frac{i_{n}}{F} & \mathrm{for}\;\mathrm{galvanostatic}\;\mathrm{operation}\\ {\left.-n\cdot J\right|}_{r=0}=0,{\left.c\right|}_{r=a}=c_{b} & \mathrm{for}\;\mathrm{potentiostatic}\;\mathrm{operation} \end{array}$$

where $c_{0}$ is initial molar concentration of lithium, n is the surface normal vector, F = 96,485.3 C/mol represents the Faraday’s constant, $i_{n}\;\left(A/m^{2}\right)$ is the surface current density of active particles and $c_{b}$ is the boundary molar concentration of lithium under constant voltage operation.

For convenience, the state of charge (SOC) is introduced to intuitively reflect the lithiation state of active material, which can be acquired by the following equation:

(5) $$SOC=c_{average}/c_{\max}=\frac{\int_{0}^{a}4\pi r^{2}cdr}{4\pi a^{3}c_{\max}/3}=\frac{3}{a^{3}c_{\max}}\int_{0}^{a}r^{2}cdr$$

When the active material is lithiated, the lithiation deformation will generate the Vegard stress, which then triggers the strain in surrounded SEI and BCC. The structural stress may endanger the mechanical integrity of the electrode particle system and ultimately results in the degradation of battery performance. Regarding the graphite particles that are considered here, it is assumed that both the active materials and SEI are linear elastic materials and thus the corresponding relationship between stress and strain can be expressed as:

(6) $$\begin{array}{l} \sigma_{r}=\frac{E}{1+v}\left(\frac{v}{1-2v}\theta +\varepsilon_{r}\right)-\frac{1}{3}\frac{E\Omega c}{1-2v}\\ \sigma_{\theta }=\frac{E}{1+v}\left(\frac{v}{1-2v}\theta +\varepsilon_{\theta }\right)-\frac{1}{3}\frac{E\Omega c}{1-2v} \end{array}$$

where $E\;\left(GPa\right)$ and v are the elastic modulus and Poisson’s ratio of the lithium compounds, respectively. $\theta =\varepsilon_{r}+2\varepsilon_{\theta }$ is the volumetric strain. $\varepsilon_{r}$ and $\varepsilon_{\theta }$ are the radial and hoop strains. The first term on the right side of Equation (6) is the mechanical elastic stress, while the latter term is related to the lithium concentration and represents the stress induced by atomic diffusion. It is noticeable that only active materials are lithiated during lithium solid-phase diffusion, and thus the above equation is only valid for active particles. For SEI, the term of diffusion induced stress in Equation (6) must be omitted.

**Figure 2:**
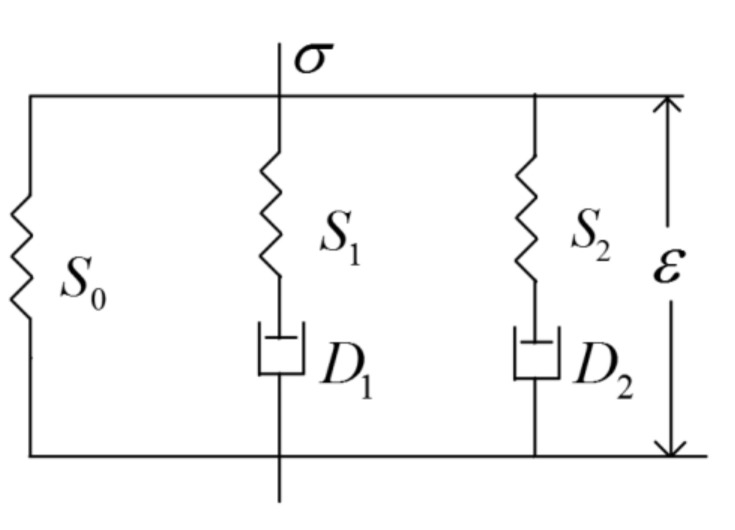
Rheological model for binder and conductive composite (BCC) composed of two Maxwell elements and a spring in parallel.

Previously, we carried out the tensile stress relaxation experiments on SA and CMC/SBR doped by Super-S carbon black at the weight ratio of 0%, 20%, 35%, 50% and 60% in 1.1-M LiPF6-EC/DMC, respectively. In terms of the evolution of normalized stress versus time, it was found all curves exhibited typical linear viscoelastic behavior (time-dependent stress reduction), but with different degrees of relaxation [32]. To this end, the deformation response of BCC is thereby characterized in term of a rheological model composed of two Maxwell elements and a spring in parallel (see Figure 2). The corresponding constitute equations are given by

(7) $$\left\{\begin{array}{l} \sigma_{vol}^{BCC}=3K^{BCC}\varepsilon_{vol}^{BCC}\\ \sigma_{dev}^{BCC}=2\int_{0}^{t}\Gamma \left(t-t^{\prime }\right)\frac{\partial \varepsilon_{dev}^{BCC}}{\partial t^{\prime }}dt^{\prime } \end{array}\right.$$

where σ and ε represent stress and strain, respectively. $K^{BCC}$ is the volume modulus and $\Gamma \left(t\right)$ is the function of relaxation modulus. The subscript ‘vol’ and ‘dev’ indicate the spherical tensors and deviator tensors, respectively. The superscript BCC means that this parameter corresponds to the binder and conductive composite. The function of the relaxation modulus can be written in terms of the Pony series:

(8) $$\Gamma \left(t\right)=G_{0}+G_{1}\exp\left(-\frac{t}{\tau_{1}}\right)+G_{2}\exp\left(-\frac{t}{\tau_{2}}\right)$$

where $G_{0}$, $G_{1}$ and $G_{2}$ represent the shear modulus of springs in the Maxwell model, respectively. $\tau_{1}$ and $\tau_{2}$ are the relaxation time values of corresponding dashpot.

According to the tensile stress relaxation curves of Super-S carbon black (SS) filled SA and CMC/SBR films [32] and Equation (8), the relaxation modulus and characteristic time are obtained by 1stOpt^®^ nonlinear regression software (7D-soft high technology incorporation, Beijing, China), and its evolution against SS content are separately listed in Table 1 and Table 2.

Neglecting the body force, the equilibrium equation in spherical coordinates can be presented as:

(9) $$\frac{d\sigma_{r}}{dr}+\frac{2}{r}\left(\sigma_{r}-\sigma_{\theta }\right)=0$$

On condition that the lithiation deformation is in the infinitesimal range, the radial strain $\varepsilon_{r}$ and hoop strain $\varepsilon_{\theta }$ can be expressed as functions of radial displacement  u:

(10) $$\varepsilon_{r}=du_{r}/dr,\varepsilon_{\theta }=u_{r}/r$$

As to the spherical symmetric structure under consideration, the radial displacement at the center of the sphere is inevitably zero in the deformation process, as the Equation (11). In the following equations, superscripts A, SEI and BCC represent the active materials, solid electrolyte interface and carbon black-filled aqueous polymer binder materials, respectively.

(11) $$u_{{}_{r}}^{A}\left|{}_{r=0}\right.=0$$

The active material–SEI interface and SEI–BCC interface meet the corresponding displacement and stress continuity conditions as presented by Equation (12):

(12) $$\begin{array}{l} u_{r}^{A}\left|{}_{r=a}=\right.u_{r}^{SEI}\left|{}_{r=a}\right.,\;\sigma_{r}^{A}\left|{}_{r=a}=\right.\sigma_{r}^{SEI}\left|{}_{r=a}\right.\\ u_{r}^{SEI}\left|{}_{r=b}=\right.u_{r}^{BCC}\left|{}_{r=b}\right.,\;\sigma_{r}^{SEI}\left|{}_{r=b}=\right.\sigma_{r}^{BCC}\left|{}_{r=b}\right. \end{array}$$

Finally, the boundary condition at the BCC surface is expressed as:

(13) $$\sigma_{r}^{BCC}\left|{}_{r=c}\right.=0$$

To numerically drive the aforementioned model in the following simulations, one can perform coupled analysis in commercial numerical software COMSOL. Here, noting that the stress within the isotropic active particle is obtained via the analogy between thermal stresses and diffusion induced stresses, the partial derivative of hydrostatic stress versus lithium concentration depends only on the material constant, i.e., $\partial \sigma_{h}/\partial c=-2E\Omega /\left[9\left(1-v\right)\right]$. The lithium concentration can thus be solved beforehand by substituting it into the governing Equation (3) and applying the corresponding initial and boundary conditions shown in Equation (4). Through the above processing, the two-way coupling between lithium diffusion and mechanical stress can be decoupled. Hence, the problem degenerates into a traditional viscoelastic problem, which is defined by Equations (6)–(13), and it can be easily solved by numerical methods everywhere [44].

## 3. Results and Discussion

### 3.1. Distribution of Lithium Concentration and Stress in Electrode Particle System

First of all, lithium diffusion and the induced stress in graphite particle, SEI film and BCC are analyzed, respectively. The viscoelasticity parameters of BCC are shown in Table 1 and Table 2 and material constants of graphite particle are listed in Table 3. The modulus and thickness of SEI are set as 1 GPa and 50 nm [6], respectively. The ratio of graphite radius, SEI thickness and BCC thickness is initially set as 1:0.01:0.1. The influence of thickness ratio and Young’s modulus on the structural stress will be discussed separately later.

**Figure 3:**
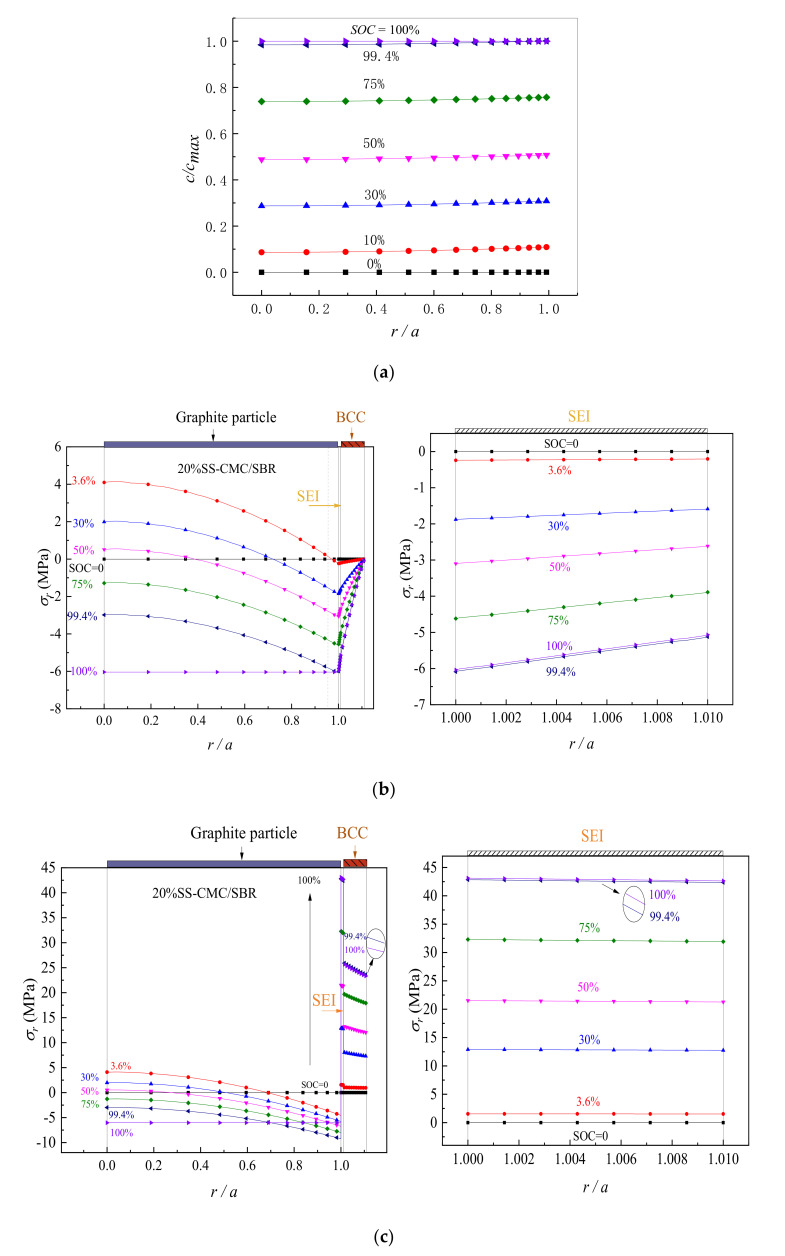
Distribution of Li concentration and stress in electrode system under different states of charge (SOC) (1C rate). (**a**) Lithium concentration in graphite particle; (**b**) radial stress; (**c**) hoop stress.

Taking 20%-SS-doped CMC/SBR as an example of BCC, we have calculated the distribution of lithium concentration in graphite particle and obtained the radial and hoop stress of the spherical electrode system under different state of charging. A charging procedure that constant current (CC) first followed by constant voltage (CV) is used to make the active material full lithiation. The first stage is galvanostatic charging (1C rate) until the lithium concentration at the boundary reaches the saturation, it then turns to the potentiostatic operation as the second stage, with the boundary concentration being $c_{b}=c_{max}$. As can be seen in Figure 3a, since the lithiation starts from the sphere surface to the inside, the lithium concentration is low at the center while high on its surface during the lithiation process, which is distributed in a radial gradient. The lithium concentration increases with the increase of SOC and eventually reaches the complete lithiation. In the meantime, Figure 3b,c shows that the stress in the graphite particle also presents nonlinear changes under the influence of structure and lithium-concentration gradient. Furthermore, as the radial stress gradient ($d\sigma_{r}/dr$) is negative and decreases from zero along the radius direction, it reaches the minimum value on the surface of the active material. Therefore, during the lithiation process, the hoop stress value gets the minimum at r=a and the maximum value exists at the sphere center. At the center of the sphere, the hoop stress is identically equal to its radial stress due to $d\sigma_{r}/dr=0$. The above results are consistent with those of Cheng et al. [45] and He et al. [44]. It can be seen from Figure 3b,c that the stress of SEI is derived from the lithiation deformation of active particle, so that the maximum stress occurs at the moment of full lithiation (SOC=100%). As for BCC, its radial stress can be determined by the continuity condition (Equation (12)) and the boundary condition (Equation (13)). The stress continuity condition is satisfied at the SEI–BCC interface, while the value in BCC surface is 0, which is in line with the result in Figure 3b. The hoop stress is different at the SEI–BCC interface due to the changes of material properties. It also presents a gradient variation similar to the hoop stress of SEI that is large near the particle center and small when far from the center. This ascribes to the structural deformation of the multilayer electrode. 

**Figure 4:**
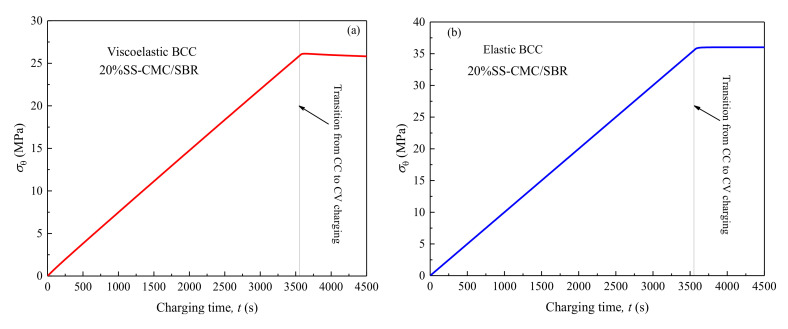
Evolution of hoop stress at solid electrolyte interface (SEI)-BCC interface (r=b) over time in the lithiation process.

It is worth noting that, influenced by the viscoelasticity of polymer binder, the peak value of hoop stress in BCC during the lithiation does not occur in the state of complete lithiation. Instead, as shown in Figure 3c, it increases in the beginning and then decreases, which is the coupling effect of electrochemical loading and material relaxation. To further illustrate the situation, Figure 4 shows that the hoop stress of BCC at the SEI–BCC interface evolves with lithiation process. Meanwhile, it compares the stress in elastic BCC with that in viscoelastic BCC. It is observed that the interface hoop stress increases linearly in the constant current (CC) charging, decreases after increasing during the potentiostatic lithiation and the peak value ($\sigma_{\theta }^{peak}$) appears in the initial stage of constant voltage (CV), which is about 26.1 MPa for viscoelastic 20% SS-CMC/SBR as seen Figure 4a. However, when the viscoelasticity of BCC is disregarded, it is clear from Figure 4b that the interface stress rises continuously as charging time increases. At the complete lithiation, it reaches the peak value, 36.0 MPa, which is about 1.4 times of that in viscoelastic case. It means that the relaxation effect of BCC cannot be ignored when evaluating the stress in composite electrode.

Noticeably, as seen in Figure 3, the peak tensile hoop stress of active particle and SEI are 4.1 MPa and 43.0 MPa, respectively, which are less compared to the tensile strength of the corresponding materials ($\sigma_{b}^{graphite}=20-100\;$ MPa [46] and $\sigma_{b}^{SEI}=45$ MPa [47]). However, above-mentioned 20% SS–CMC/SBR in the lithiation has subjected to very high tensile deformation and $\sigma_{\theta }^{peak}$ is up to 165% larger compared to the tensile strength of BCC (15.8 MPa [32]). It would lead to circumferential cracking in BCC and affect the structural integrity. Therefore, it is necessary to explore the effects of aqueous binder type, conductive agent content, SEI properties and charging condition on the peak stress in BCC.

### 3.2. Effects of Carbon Black Contents and Polymer Type on Peak Stress in BCC

**Figure 5:**
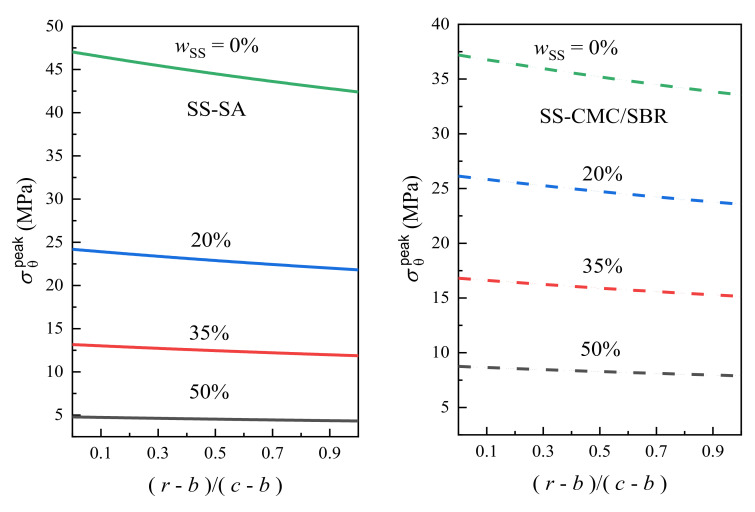
Effect of carbon black contents ($w_{SS}$) and polymer type on peak hoop stress ($\sigma_{\theta }^{peak}$) in BCC.

Next comes the discussion of $\sigma_{\theta }^{peak}$ in BCC with the different mass fractions of SS carbon black. The lithiation condition remains unchanged and the simulation results of peak hoop stress are shown in Figure 5.

As seen in Figure 5, with the increase of SS loading from 0% to 50%, the hoop stress in BCC of both the SA and CMC/SBR matrices decreases and its distribution gets more uniforms. For SS-SA, the value of $\sigma_{\theta }^{peak}$ in BCC decreases by 90%, which is from the initial 47.0 to 4.8 MPa. As to the SS-CMC/SBR, the peak stress decreases by 76%, from 37.2 to 8.8 MPa. The engineering stress-strain (σ ~ε) curves of neat CMC/SBR and SA-based BCC films display the features of brittle polymers, however, when the carbon black is added, it gradually tends to show more features of ductile polymers [32]. As such, the variation of $\sigma_{\theta }^{peak}$ in BCC is mainly a consequence of the decline of elastic modulus caused by the transition from brittleness to ductility and the rapid structure relaxation. Remarkably, virgin SA has sustained a much higher hoop stress compared to CMC/SBR. Nevertheless, $\sigma_{\theta }^{peak}$ in SS-SA rapidly decreases as SS loading increases from 20% to 50%, and it is gradually lower than that in SS-CMC/SBR at the same content of carbon black [32]. This is ascribed to the effect of conductive agent addition on the reduction of stiffness and relaxation time that is relatively noticeable for SA matrix as seen in Table 1 and Table 2.

**Figure 6:**
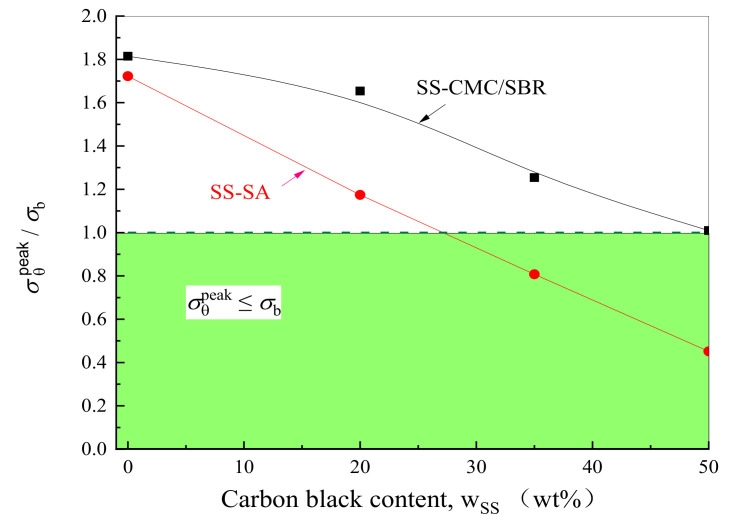
Mechanical integrity of BCC with different carbon black contents and aqueous polymer.

Although adding conductive carbon black into the binder contributes to the reduction of structural stress, the previous mechanical experiment revealed that the increase of SS contents also weakens the tensile strength ($\sigma_{b}$) of water soluble polymer-based composites [32]. Hence, in order to further analyze the mechanical integrity of BCC under different addition amounts of carbon black, Figure 6 exhibits the variation of the ratio of $\sigma_{\theta }^{peak}$ in BCC to its $\sigma_{b}$ against SS mass fraction. From figure, it can be seen that with the increased concentration of carbon black, the magnitudes of $\sigma_{\theta }^{peak}/\sigma_{b}$ gradually switch from being greater than or equal to 1 to less than 1. Such a transition manifests that the higher the SS content, the smaller the ratio of BCC stress to its strength—and the less prone the polymer composite is to become damaged in the circumferential direction, which is more conducive to ensuring the mechanical integrity of BCC in the electrochemical cycling process. Importantly, the threshold values of carbon black content are 27 wt% and 50 wt% for SS-SA and SS–CMC/SBR, respectively. Further, at the same loading of conductive carbon black, SS-SA exhibits much better resistance to tensile failure compared to SS-CMC/SBR. The increase of carbon black helps not only to enhance the structure stability of BCC, but to enrich the conductive network in BCC and further improve its electrical properties. Considering that the excess inactive material would decrease the specific capacity of cell, 50 wt% of carbon black is a compromise constitute content in terms of the comprehensive mechanical and electrical properties, which is in accordance with the 1:1 ratio of carbon black and polymer binder in commercial lithium batteries.

### 3.3. Effect of SEI Modulus and Thickness on Peak Stress in BCC

The above research demonstrates that the tensile hoop stress in the electrode particle system is the main cause for the damage of polymer binder and conductive composites. Zhang et al. reported that SEI film exhibits a quite large heterogeneity in the measured modulus which varies from 10 MPa to 10 GPa [48]. How does the variation in both SEI modulus and thickness impact the structure integrity of BCC? The following discussion aims to elucidate this problem by comparing the peak stress in BCC under different SEI parameters.

**Figure 7:**
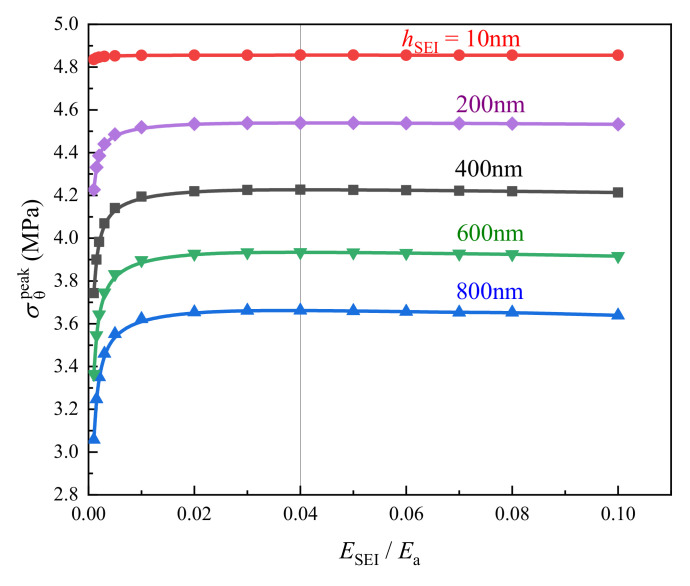
Effects of modulus ($E_{SEI}$) and thickness of SEI ($h_{SEI}$) on peak hoop stress ($\sigma_{\theta }^{peak}$) in BCC.

**Figure 8:**
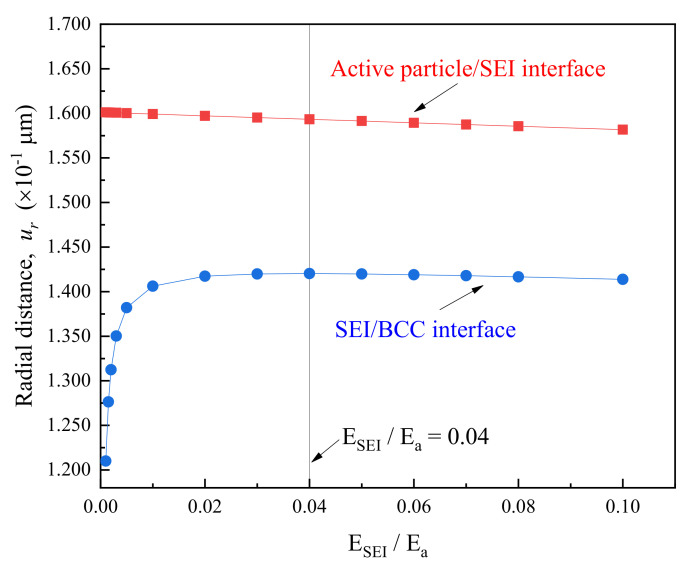
Effect of relative modulus ($E_{SEI}/E_{a}$) of SEI to active particle on interfacial distance.

The peak hoop stress in BCC was simulated for the composite electrode charged by the mixed mode (initially galvanostatic followed potentiostatic lithiation) and it varied with the change of SEI stiffness (as shown in Figure 7). The calculation showed that $\sigma_{\theta }^{peak}$ in BCC with the constant film thickness initially enlarges and then decreases as the value of $E_{SEI}/E_{a}$ increases from 0.001 to 0.1 (10–1000 MPa). When the relative elastic modulus of SEI against active particle was about 0.04, it brings about the BCC with the highest $\sigma_{\theta }^{peak}$ for a group of samples. This was because—for extremely softer SEI ($E_{SEI}\leq 100$ MPa)—the stiffening of SEI could not actually enhance the structural constraints on the active particle, of which elastic modulus was larger than 100 times of SEI in this case, leading to only slight effect on lithiation-induced deformation. However, it would substantially give rise to the increased tensile radial deformation of relaxed BCC attributed to the comparative stiffness of SEI film, as seen in Figure 8. Additionally, further hardening of SEI could effectively hamper the deformation of active particle upon lithiation/delithiation, contributing to the linear decline in radial strain of both SEI and BCC. Therefore, the peak hoop stress in BCC became gradually weakened and the influence was more obvious for the thicker SEI as shown in Figure 7. In another hand, if the BCC could endure the tensile loading at critical relative modulus of SEI respective to active material, the variation in physical properties of passivated layer caused by electrochemical cycling would not endanger the binder and conductive composite. In addition, it is easy to understand that the magnitudes of $\sigma_{\theta }^{peak}$ in BCC decay with the increased SEI thickness owing to the stronger constraint on the deformation of electrode system. Previous literature indicated that the factors influencing the material parameters of SEI films were quite complex [49]. Their thickness and mechanical properties were closely related to the active materials, liquid electrolyte and its additives, as well as the charging and discharging environment, etc. Therefore—based on the comprehensive optimal design of the electrode material and lithiation conditions—a suitable SEI layer appears also important to maintain mechanical stability BCC in the composite electrode during electrochemical process. It was noted that BCC sustained a peak hoop stress of around 4.86 MPa at $h_{SEI}=10\;$nm, which was perceivably higher than that at $h_{SEI}\geq 200$ nm. In another word, neglecting SEI would markedly mislead the structure integrity of polymer binder and conductive composite.

### 3.4. Effects of Lithiation Rate and Thickness of BCC on its Peak Stress

The previous works demonstrate clearly that CV or CC charging at a higher rate would increase the concentration inhomogeneity, resulting in larger stress in the active layer [50] and SEI [51]. Due to the relaxation nature of polymer binder, it is not clear how the charging rate affects the peak stress in BCC. In addition, the variation in the thickness of binder and conductive film can change the constraint on the composite electrode; the corresponding influence on the mechanical integrity of viscoelastic BCC is also worthy of in-depth investigation. Herein, we still take 50% SS-SA as an example to analyze the evolution of $\sigma_{\theta }^{peak}$ in BCC with various thickness and lithiation rate.

**Figure 9:**
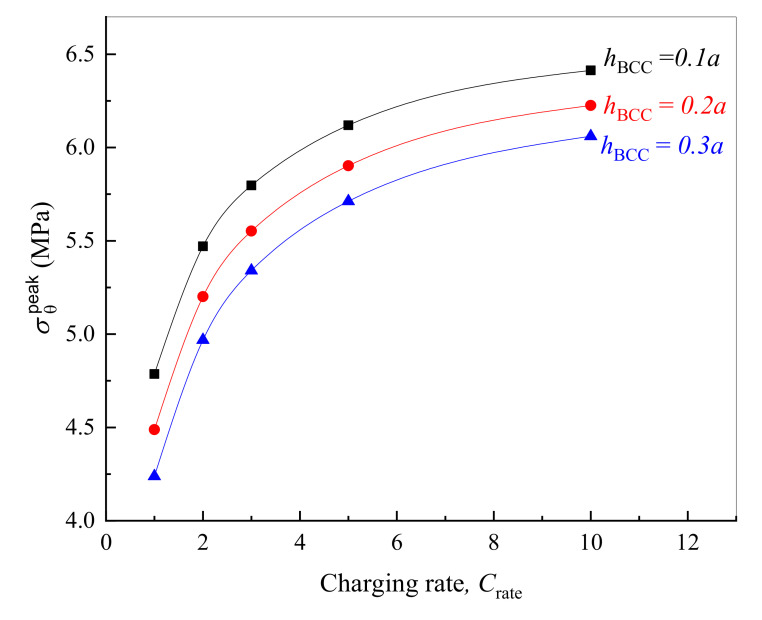
Effects of lithiation rate and BCC thickness ($h_{BCC}$) on its peak stress.

As far as the given multilayer electrode structure, Figure 9 demonstrates that the higher the charging rate is, the greater the peak hoop stress in BCC will be during electrode lithiation, and the stress gradually tends to be constant as lithiation goes on. There are two major reasons. On one hand, the lithium concentration on the surface of active particles is close to saturation when inserted by lithium with a high current rate under galvanostatic operation. It thus converts to the potentiostatic lithiation earlier. On the other hand, the faster the lithiation is, the slower the BCC relaxation is—and the stiffer the polymer composite exhibits, which results in a larger $\sigma_{\theta }^{peak}$ of the structure. As expected, a thinner BCC displays higher peak stress owing to the decrease in the cross-sectional area subjected to loading as seen in Figure 9.

It is clear from the above that increasing the thickness or reducing the current intensity of lithiation can make the peak stress in BCC lower than its tensile strength. These methods are both beneficial to enhancing the rupture resistance of bonding material. However, a thicker BCC means a relatively lower amount of active substance in the electrode material, leading to a reduction in the specific energy density of the battery. As such, the content of binder and conductive agent in practical design should be as small as possible on the premise of ensuring the structural integrity of the electrode particle system.

## 4. Conclusions

- Higher tensile hoop stress may occur for polymer binder and conductive composite (BCC) in the electrode during charging process. As a result, the circumferential cracks primarily initiated in these inactive materials rather than other electrode components. Further, mechanical damage did not take place at complete lithiation on account of the polymer viscoelasticity and would be greatly misled if neglecting the effect of SEI.
- With the increase of conductive agent content, the stress level of the bonding system decreased significantly under the electrochemical operation. To ensure the mechanical integrity of the graphite anode with aqueous binders, the minimum mass concentration of carbon black added in SA and CMC/SBR should be 27% and 50%, respectively. Moreover, SA-based composite exhibited much better rupture resistance compared to the counterparts at the same content of conductive agent.
- On the basis of the evolution of peak stress in multilayer spherical structures, a robust composite electrode may be obtained by: (1) reducing the elastic modulus of the SEI at least to less than that of the BCC; (2) lithiation at lower rate; (3) increasing the both BCC and SEI as much as possible in the context of meeting cell capacity requirements; (4) improving the tensile strength of BCC up to larger than the peak hoop stress in BCC at critical relative stiffness of SEI to active particle.

## Figures and Tables

**Table 1:** Viscoelasticity parameters of sodium alginate (SA) with different carbon black contents.

SS Contents (wt%)	*G*_0_ (MPa)	*G*_1_ (MPa)	*τ*_1_ (min)	*G*_2_ (MPa)	*τ*_2_ (min)
0	320.3	195.3	51.85	132.67	2.1
20	163.6	140.11	28.26	94.9	1.6
35	84.2	103.87	24.13	*69.24*	1.33
50	27.9	57.56	19.57	35.04	1.18

**Table 2:** Viscoelasticity parameters of sodium carboxymethyl cellulose (CMC)/styrene–butadiene rubber (SBR) with different carbon black contents.

SS Contents (wt%)	*G*_0_ (MPa)	*G*_1_ (MPa)	*τ*_1_ (min)	*G*_2_ (MPa)	*τ*_2_ (min)
0	247.4	110.44	295.6	73.62	3.18
20	176.2	74.42	253.16	56.03	1.89
35	104.2	68.06	174.45	45.38	1.72
50	51.6	43.81	130.55	21.8	0.59

**Table 3:** Material parameters of graphite particles [21].

Poisson’s ratio	0.3
Young’s modulus (GPa)	10.0
Partial molar volume of Li (m^3^/mol)	3.17 × 10^−6^
Li diffusion coefficient (m^2^/s)	4.9 × 10^−14^
Saturated Li concentration (mol/m^3^)	3.05 × 10^4^
